# Hypochlorite-induced oxidation promotes aggregation and reduces toxicity of amyloid beta 1-42

**DOI:** 10.1016/j.redox.2023.102736

**Published:** 2023-05-13

**Authors:** Noralyn B. Mañucat-Tan, Ashfaq Chowdhury, Rodrigo Cataldi, Rafaa Zeineddine Abdullah, Janet R. Kumita, Amy R. Wyatt

**Affiliations:** a Flinders Health and Medical Research Institute, College of Medicine and Public Health, Flinders University, SA, Australia, 5048; b Yusef Hamied Department of Chemistry, University of Cambridge, Lensfield Road, Cambridge, CB2 1EW, UK; c Illawarra Health and Medical Research Institute and School of Biological Sciences, University of Wollongong, NSW, Australia, 2500; d Department of Pharmacology, University of Cambridge, Tennis Court Road, Cambridge, CB2 1PD, UK

**Keywords:** protein misfolding, Alzheimer's Disease, oxidative stress, Renewable energy, inflammation, post-translational modification

## Abstract

Exacerbated hypochlorite (OCl^−^) production is linked to neurodegenerative processes, but there is growing evidence that lower levels of hypochlorite activity are important to protein homeostasis. In this study we characterise the effects of hypochlorite on the aggregation and toxicity of amyloid beta peptide 1–42 (Aβ_1-42_), a major component of amyloid plaques that form in the brain in Alzheimer's disease. Our results demonstrate that treatment with hypochlorite promotes the formation of Aβ_1-42_ assemblies ≥100 kDa that have reduced surface exposed hydrophobicity compared to the untreated peptide. This effect is the result of the oxidation of Aβ_1-42_ at a single site as determined by mass spectrometry analysis. Although treatment with hypochlorite promotes the aggregation of Aβ_1-42_, the solubility of the peptide is enhanced and amyloid fibril formation is inhibited as assessed by filter trap assay, thioflavin T assay and transmission electron microscopy. The results of *in vitro* assays using SH-SY5Y neuroblastoma cells show that pre-treatment of Aβ_1-42_ with a sub-stoichiometric amount of hypochlorite substantially reduces its toxicity. The results of flow cytometry analysis and internalisation assays indicate that hypochlorite-induced modification of Aβ_1-42_ reduces its toxicity via at least two-distinct mechanism, reducing the total binding of Aβ_1-42_ to the surface of cells and facilitating the cell surface clearance of Aβ_1-42_ to lysosomes. Our data is consistent with a model in which tightly regulated production of hypochlorite in the brain is protective against Aβ-induced toxicity.

## Graphical abstract

**Figure:**
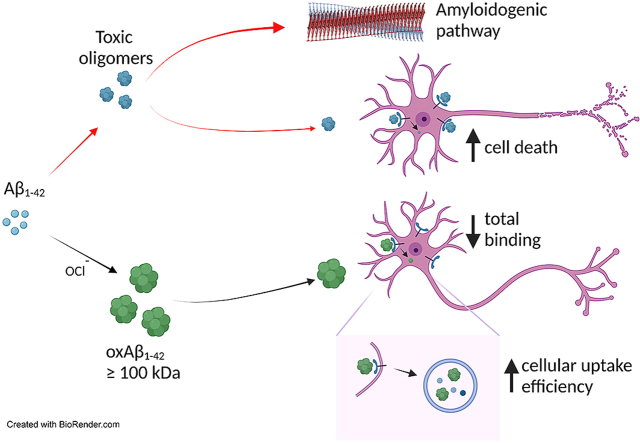

## 1 Introduction

Alzheimer's disease (AD) is the leading cause of dementia and is estimated to affect close to 50 million people worldwide. Despite a century of research into the condition, effective therapies have not been developed and there are still many unknowns regarding the pathogenesis of the disease. Primary hallmarks of AD include the accumulation of amyloid beta (Aβ) peptide and hyperphosphorylated tau that form extracellular amyloid plaques and intracellular neurofibrillary tangles, respectively. A substantial body of evidence supports the idea that neuroinflammation is the third core pathology in AD [1], however, neuroinflammation is also important to tissue repair and a normal response to tissue damage and ageing. Therefore, it is imperative to characterise the inflammatory mechanisms that promote pathologic versus protective responses in the ageing brain.

The relationship between the accumulation of Aβ and neuroinflammation is complicated. For example, activation of brain-resident immune cells is a fundamentally important defence mechanism that facilitates the clearance of neurotoxic Aβ from biological fluids [2]. On the other hand, activation of immune cells exacerbates pro-inflammatory responses and promotes the generation of reactive oxygen species that can contribute to tissue damage [[3], [4], [5]]. It is likely that in AD, neuroinflammation plays a multifaceted role that changes over time as the severity of the disease progresses. Consistent with this idea, clinical trials of anti-inflammatory drugs have not convincingly demonstrated prophylactic or therapeutic efficacy in AD patients [5].

In AD, oxidative modification of Aβ occurs but the significance of this is not yet clear [6,7]. Depending on the experimental system used, oxidative modification of Aβ can either promote or supress its neurotoxicity *in vitro* [6,[8], [9], [10], [11], [12], [13], [14]]. Many relevant studies have used metal-catalysed systems to study the effect of oxidation on Aβ, yet hydroxyl radicals (^•^OH; produced by metal-catalysed Fenton chemistry) are just one of many biological oxidants that are generated during inflammation. A growing body of work has demonstrated that the functional consequences of protein oxidation are dependent on both the oxidant and the protein identity [[15], [16], [17], [18]], as such there is a need to assess the specific effects of individual biological oxidants on the functions of Aβ.

Several lines of evidence support the conclusion that Aβ is an endogenous target of the inflammatory oxidant hypochlorite (OCl^−^; generated by the enzyme myeloperoxidase). For example, hypochlorite-modified proteins have been shown to accumulate in the brain of AD patients and myeloperoxidase reactive microglia are found co-localised with Aβ in senile plaques [19]. To a lesser extent 3-chlorotyrosine, a specific marker of hypochlorite-induced protein modification, is also measured in cognitively normal brain tissue [19]. It has previously been reported that hypochlorite-induced modification inhibits the fibrillar aggregation of Aβ_25-35_ (a short A*β* fragment) [20], but detailed characterisation of the effect of hypochlorite on abundant AD-associated Aβ forms (typically 40–42 amino acids in length) has not previously been carried out. To address an important gap in knowledge, this study examines the effect of hypochlorite on the aggregation and toxicity of Aβ_1-42_ *in vitro.*

## 2 Results

### 2.1 Incubation with sodium hypochlorite (NaOCl) induces the formation of soluble oligomeric Aβ_1-42_ assemblies

**Fig. 1:**
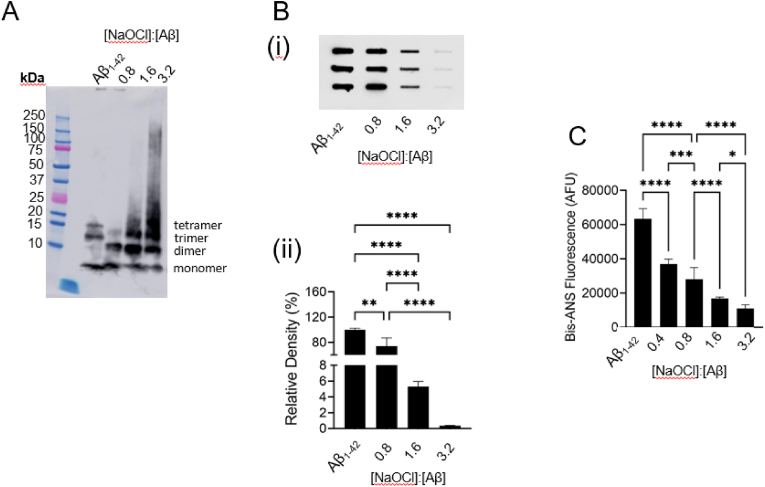
**Effect of NaOCl on the solubility and surface hydrophobicity of Aβ**_**1-42**_. (**A**) Western blot image showing Aβ_1-42_ following treatment with NaOCl in PBS overnight at ambient room temperature. The molar ratio of NaOCl to Aβ is indicated for each sample. Aβ_1-42_ samples were separated on a 10–20% tris-tricine gel and transferred to PVDF membrane. The membrane was probed using monoclonal anti-Aβ_1-42_ (WO2) and a relevant secondary antibody before visualisation by enhanced chemiluminescence. (**B**) (i) Western blot image showing Aβ_1-42_ retained on a cellulose acetate membrane after filter trap assay, following treatment of Aβ_1-42_ with NaOCl as described in (A) (ii) Corresponding chart shows the relative density of Aβ_1-42_. The data points are means (*n* = 3; ±SD) **p < 0.01; ****p < 0.0001; One-way ANOVA, Tukey's test). (**C**) Bis-ANS fluorescence *(Excitation =* 360 nm*, Emission =* 502 nm*)* of Aβ_1-42_ samples after treatment with NaOCl as in (A). The data are the mean (n = 10; ±SD). **** Control samples are Aβ_1-42_ incubated as described, but in PBS alone.

In the presence of SDS, Aβ_1-42_ predominately migrates as a monomer, trimers or tetramers as assessed by Western blot analysis (Fig. 1A). Incubation of Aβ_1-42_ with a sub-stoichiometric amount of NaOCl promoted the formation of SDS-resistant Aβ_1-42_ dimers and concomitantly reduced the abundance of Aβ_1-42_ trimers and tetramers (Fig. 1A). Treatment of Aβ_1-42_ with higher concentrations of NaOCl, induced the formation of dimers and higher order Aβ_1-42_ assemblies up to ∼150 kDa (Fig. 1A). To demonstrate the effect of NaOCl on the solubility of Aβ_1-42_ under detergent-free conditions, a filter trap assay was performed. The results show, that with increasing NaOCl concentrations, there is a decrease in the amount of insoluble Aβ_1-42_ bound to the cellulose acetate membrane (Fig. 1Bi). Densitometry analysis showed that NaOCl increased the solubility of Aβ_1-42_ in phosphate buffered saline (PBS), pH 7.4 in a dose-dependent manner (Fig. 1Bii). Compared to Aβ_1-42_ incubated in PBS overnight incubation at ambient room temperature, co-incubation with a 1.6-fold molar excess of NaOCl resulted in a 95% reduction in insoluble Aβ_1-42_ detected by filter trap assay. Analysis of the Aβ_1-42_ preparations by BisANS assay showed that incubation with NaOCl has the general effect of reducing the surface exposed hydrophobicity of Aβ_1-42_ in solution (Fig. 1C). This effect was dose-dependent and there was a substantial decrease in BisANS fluorescence even when NaOCl was present at a sub-stoichiometric concentration compared to Aβ_1-42_ (Fig. 1C). In control experiments we confirmed that NaOCl at the concentrations used did not influence BisANS fluorescence (Sup Fig. 1A).

**Fig. 2:**
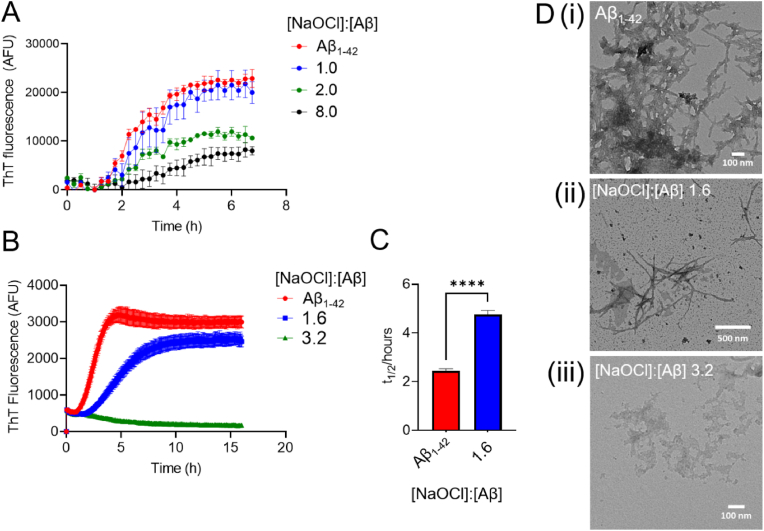
**Effect of NaOCl on Aβ**_**1-42**_**amyloid fibril formation, as assessed by ThT assay and TEM** (**A**) Graph shows the ThT fluorescence of 5 μM Aβ_1-42_ incubated in the presence or absence of NaOCl in PBS at 28 °C with orbital shaking in a Clariostar platereader. All samples contained 25 μM ThT. Data is the mean (n = 4; ±SD) of the background adjusted ThT fluorescence. (**B**) Graph shows the ThT fluorescence of 4 μM Aβ_1-42_, pre-treated in the presence or absence of NaOCl in PBS for 1 h at RT and then supplemented with excess L-Met. Aβ_1-42_ was then incubated in the presence of 25 μM ThT at 37 °C with orbital shaking in Clariostar platereader overnight. Data is mean (n = 3; ±SD) of the background adjusted ThT fluorescence. (**C**) Chart shows the half-time (t_1/2_) values for ThT fluorescence for samples shown in (B). Data is mean (n = 3; ±SD) ****p < 0.0001, unpaired *t*-test. (**D**) Corresponding TEM images for samples taken at the end point of the assay shown in (B).

Consistent with the results obtained using the filter trap assay, incubation with NaOCl reduced amyloid-associated thioflavin T (ThT) fluorescence in a dose-dependent manner (Fig. 2). Under conditions that promote amyloid fibril formation, a 2-fold molar excess of NaOCl was required to significantly suppress the amyloid-associated ThT fluorescence over a 7-hr time course (Fig. 2A). There was also a modest effect on extending the lag phase of Aβ_1-42_ aggregation compared to control Aβ_1-42_ incubated in the absence of NaOCl***.*** There was negligible effect of NaOCl on ThT fluorescence at the concentrations used in these assays (Sup. Fig. 1B). Pre-treating Aβ_1-42_ with NaOCl prior to the ThT assay increased the effect of NaOCl on amyloid-associated ThT fluorescence (Fig. 2B). Under these conditions, pre-treatment of Aβ_1-42_ with a 1.6-fold molar excess of NaOCl significantly delayed the increase in amyloid-associated ThT fluorescence (Fig. 2C). Additionally, pre-treatment of Aβ_1-42_ with a 3.2-fold molar excess of NaOCl prevented amyloid-associated ThT fluorescence over the 15 h of the assay (Fig. 2B). In separate experiments we confirmed that l-methionine (L-Met; used to quench the reaction between NaOCl and Aβ_1-42_) did not influence the aggregation of Aβ_1-42_ under the conditions used (Sup. Fig. 1C). Analysis of the end-point samples obtained following the ThT assay by transmission electron microscopy (TEM) confirmed that the formation of amyloid fibrils was markedly reduced by treatment of the peptide with NaOCl (Fig. 2Di-iii). Instead, incubation of Aβ_1-42_ with NaOCl promoted the formation of smaller amorphous Aβ_1-42_ assemblies (Fig. 2D ii-iii).

**Fig. 3:**
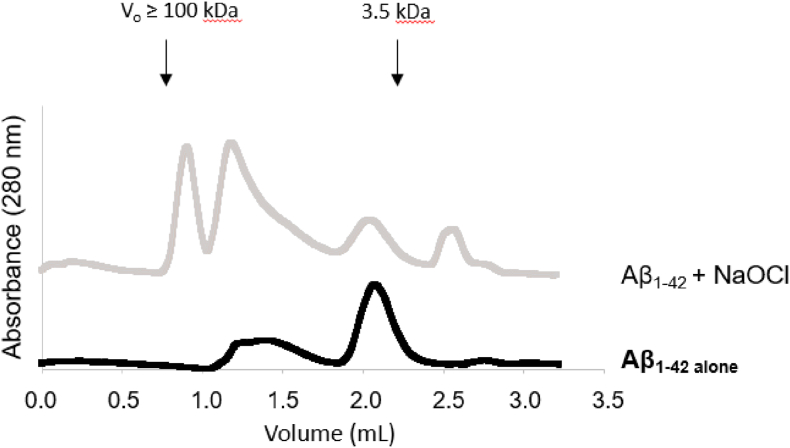
**Effect of NaOCl on Aβ**_**1-42**_**aggregation, as assessed by size exclusion chromatography**. Chromatograms show the elution of Aβ_1-42_ separated on a Superdex 75 Increase 3.2/300 column at a flow rate of 0.05 ml/min in PBS as measured using the absorbance at 280 nm. Black line shows the elution of Aβ_1-42_ in PBS after 1 h incubation at ambient room temperature. Grey line shows the elution of Aβ_1-42_ after treatment with a 2-fold molar excess of NaOCl in PBS for 1 h at ambient room temperature.

To gain insight regarding the size of the soluble Aβ_1-42_ assemblies induced by incubation with NaOCl, size exclusion chromatography was performed. In the absence of NaOCl treatment, a small fraction of the peptide eluted as low-order oligomers ≤20 kDa, but the majority of the peptide eluted corresponding to the expected size of the Aβ_1-42_ monomer (4.5 kDa) (Fig. 3). Following treatment with NaOCl at a 2-fold molar excess, a decrease in the monomer fraction is observed and the predominate peaks corresponded to approximate molecular masses of 20 kDa and ≥100 kDa (exclusion limit) (Fig. 3), which is consistent with the formation of Aβ_1-42_ tetramers and higher order assemblies in solution.

### 2.2 The reaction of Aβ_1-42_ with NaOCl results in the generation of a specific oxidised product

**Fig. 4:**
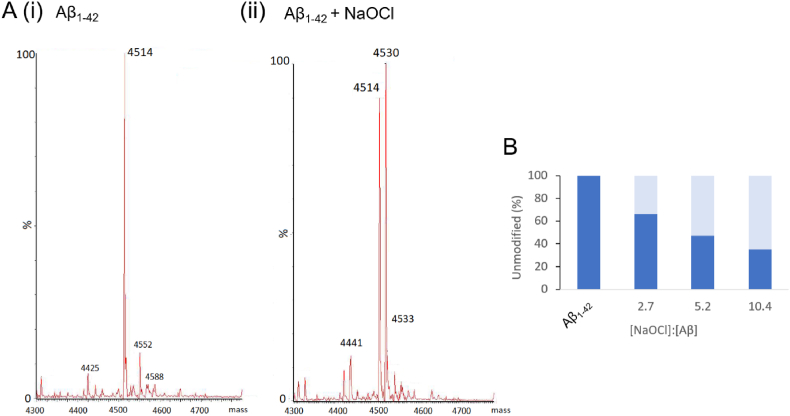
**Effect of NaOCl on the oxidation of Aβ**_**1-42**_. (**A**) Maximum entropy mass spectrum of (i) control Aβ_1-42_ or (ii) Aβ_1-42_ incubated with a 5.2-fold molar excess of NaOCl in PBS for 1 h at 4 °C. Masses are shown in daltons. (**B**) Chart shows the amount of unmodified Aβ_1-42_ (4515 Da) remaining after treatment with NaOCl in PBS for 1 h at 4 °C. The molar ratio of [NaOCl]:[Aβ] used is indicated. Data is expressed relative to a control sample that was incubated in the absence of NaOCl.

To determine the extent to which Aβ_1-42_ was modified by NaOCl under the conditions used in this study, mass spectroscopy analysis was performed. The results show that the treatment of Aβ_1-42_ with NaOCl results in the conversion of unmodified Aβ_1-42_ (MW = 4514 Da; Fig. 4Ai) to a singly oxidised species (MW = 4530 Da; Fig. 4Aii). This occurs in a dose-dependent manner, whereby at a 5-fold molar excess of NaOCl, around half of the peptide in the preparation was oxidised (Fig. 4B). These results are consistent with the specific oxidation of Met35 of Aβ_1-42_, which is highly susceptible to oxidation compared to other amino acids in the sequence of Aβ_1-42_ [20].

### 2.3 Treatment with NaOCl potently reduces the neurotoxicity of Aβ_1-42_*in vitro*

**Fig. 5:**
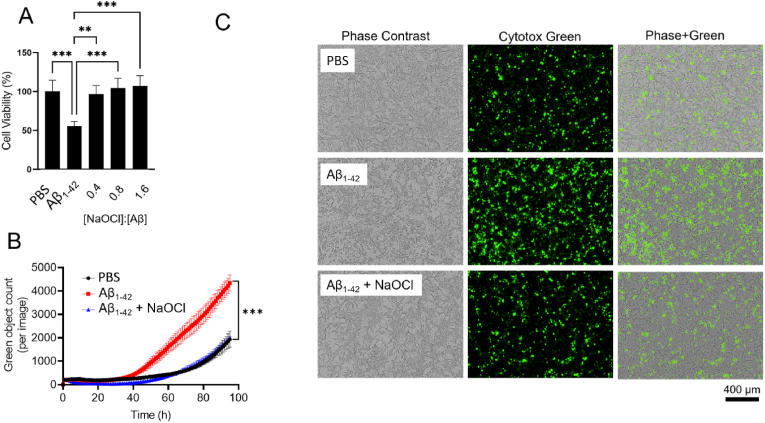
**Effect of NaOCl on the toxicity of Aβ**_**1-42**_**against SH-SY5Y neuroblastoma cells*****in vitro***. (**A**) Chart shows the percent cell viability of SH-SY5Y cells as measured by MTS assay following treatment with 10 μM Aβ_1-42_ or vehicle (PBS) for 48 h. Aβ_1-42_ was pre-incubated in Ham's/F12 containing NaOCl at the molar ratios indicated for 4 days at 4 °C and the reaction quenched using excess l-methionine (L-Met) prior to the assay. The data are the mean cell viability (*n* = 3; ±SD) and are expressed relative to the vehicle control **p < 0.01; ***p < 0.001; One-way ANOVA, Tukey's test. **(B)** Graph shows the green object count, indicative of dead cells, as measured using an Incucyte live cell imager. Cells were cultured in the presence of 15 μM Aβ_1-42_ or vehicle (PBS). Aβ_1-42_ (100 μM) was pre-incubated in Ham's/F12 ± 80 μM NaOCl for 2 h at ambient room temperature prior to the assay. **(C)** Representative images taken at 72 h for the assay shown in (B). (For interpretation of the references to colour in this figure legend, the reader is referred to the Web version of this article.)

Although there is controversy regarding the precise species responsible for Aβ-induced neurotoxicity, it is generally accepted that the aggregation of Aβ underpins its neurotoxic effects. Considering that our data shows that NaOCl-induced modification promotes the formation of soluble, aggregated Aβ_1-42_ assemblies, the toxicity of NaOCl-treated Aβ_1-42_ was assessed *in vitro*. Compared to a control sample of Aβ_1-42_ that was incubated in the absence of NaOCl, pre-treatment of Aβ_1-42_ with sub-stoichiometric concentrations of NaOCl abolished the neurotoxicity of Aβ_1-42_ against SH-SY5Y neuroblastoma cells *in vitro*, as assessed using MTS assay (Fig. 5A) and Cytotox green assay (Fig. 5B and C). Analysis of vehicle only control cells supplemented with NaOCl and L-Met confirmed that the incipient chemicals had no effect on cell viability (Sup. Fig. 2A and B). As assessed using an Incucyte assay, cell death after 72 h was approximately halved by pre-incubation of Aβ_1-42_ with a substoichiometric amount of NaOCl (Fig. 5B).

### 2.4 Treatment with NaOCl reduces the binding of Aβ_1-42_ to SH-SH5Y cells

**Fig. 6:**
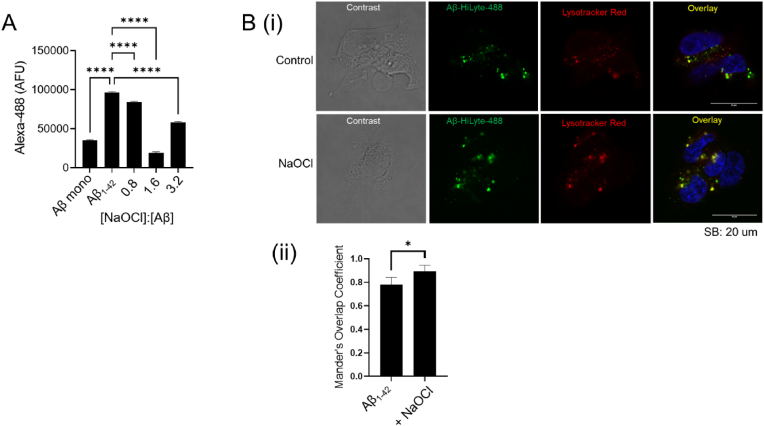
**Effect of NaOCl on the cell-surface binding and internalisation of Aβ**_**1-42**_***in vitro***. (**A**) Chart shows the Alexa fluor 488 fluorescence of SH-SY5Y cells following incubation with with 5 μM biotinylated Aβ_1-42_. Prior to incubation with the cells, biotinylated Aβ_1-42_ was pre-incubated with 0–160 μM NaOCl in PBS at ambient temperature overnight. Biotinylated Aβ_1-42_ monomer was obtained directly from frozen storage. Aβ_1-42_ is the control incubated in PBS in the absence of NaOCl. The molar ratio of NaOCl to Aβ in the remaining samples is indicated on the chart. The data are means (*n* = 3; ±SD) ****p < 0.0001; One-way ANOVA, Tukey's test). *Asterisks* denotes significant difference between samples and control. **B(i)** Representative confocal images of SH-SY5Y cells following incubation with Aβ_1-42_-Hilyte-488 (green), Lysotracker Red DND-99 (red) and Hoechst 33452 (blue). Aβ_1-42_-Hilyte-488 (50 μM) was pre-incubated ± 40 μM NaOCl in Ham's/F12 for 24 h at RT. SH-SY5Y cells were then cultured in the presence of Aβ_1-42_-Hilyte-488 (2.5 μM) for 24 h using standard culture conditions. Cells were stained with Lysotracker Red DND-99 and Hoechst 33452 for 30 min at 37 °C, prior to being fixed and imaged using a Zeiss LSM880 confocal microscope. Scale bar is 20 μm. (**ii**) The overlap coefficient between Aβ (green) in the lysotracker stained vesicle (red) was analyzed using the ZEN Black software and the Mander's coefficient calculated. The data is mean (n = 5; ±SD). *p < 0.05; unpaired student's t-test). (For interpretation of the references to colour in this figure legend, the reader is referred to the Web version of this article.)

The binding of Aβ_1-42_ to receptors on the surface of neurons initiates a cascade of responses that contribute to neurodegeneration (reviewed in [[21], [22], [23]]). To investigate the effect of hypochlorite-induced modification on the cell surface binding of Aβ_1-42_ to SH-SY5Y cells, the binding of NaOCl-modified Aβ_1-42_ preparations to the cell surface was measured using flow cytometry (Fig. 6A). The results show that treatment with NaOCl has the general effect of reducing Aβ_1-42_-associated fluorescence, indicative of a reduction in binding to the surface of SH-SY5Y cells compared to Aβ_1-42_ pre-treated with PBS alone (Fig. 6A). The effect was relatively modest when Aβ_1-42_ was treated with sub-stoichiometric concentrations of NaOCl, however, Aβ_1-42_-associated fluorescence was reduced by around 70% when Aβ_1-42_ was pre-treated with NaOCl at a 1.6-fold molar excess. The amount of Aβ_1-42_ binding measured under these conditions was similar to monomeric Aβ_1-42_ that is not potently toxic to cells. When a higher concentration of NaOCl was used to pre-treat Aβ_1-42_, there was an increase in Aβ_1-42_-associated cell surface fluorescence, however, this value was still less than the fluorescence resulting from incubation of the cells with a control Aβ_1-42_ preparation. Quality control analysis was performed to confirm that treatment with NaOCl did not reduce the binding of biotinylated Aβ_1-42_ to streptavidin, as used in cell binding assays (Sup Fig. 3A). Additionally, biotinylation did not affect the propensity of Aβ_1-42_ to form SDS-resistant dimers following treatment with NaOCl (Sup Fig. 3B).

### 2.5 NaOCl-treated Aβ_1-42_ is more efficiently internalised to lysosomes compared to neurotoxic Aβ_1-42_ preparations

Considering that treatment with NaOCl reduces but does not abolish the binding of Aβ_1-42_ to SH-SY5Y cells, we next performed experiments to elucidate the fate of NaOCl-modified Aβ_1-42_ following its binding to the cell surface. In these experiments, Aβ_1-42_ was pre-treated with NaOCl at a 0.8 molar ratio [NaOCl]:[Aβ], which only results in a modest reduction in binding of the peptide to the cell surface (∼15%) compared to untreated Aβ_1-42_ (Fig. 6A). Using confocal microscopy it was observed that NaOCl-treated Aβ_1-42_ is internalised to endocytic vesicles and calculation of the Mander's overlap coefficient suggests that internalisation to lysosomes was around 10% more efficient for NaOCl-treated Aβ_1-42_ compared to untreated Aβ_1-42_ (Fig.6Bi and ii). Under the conditions used, there were no substantial reduction in Hilyte-488 fluorescence as a result of pre-treatment of the labeled peptide with NaOCl (Sup. Fig. 4).

**Fig. 7:**
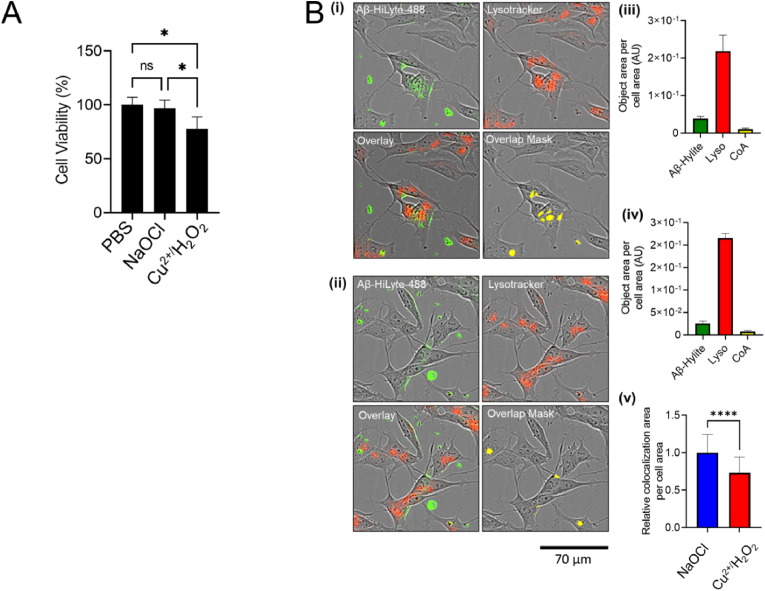
**Effect of oxidizing system on Aβ**_**1-42**_**neurotoxicity and internalisation*****in vitro**.* (**A**) Chart shows the percent cell viability of SH-SY5Y cells as measured by MTS assay following 48 h treatment with 20 μM Aβ_1-42_. Aβ_1-42_ was pre-incubated with NaOCl or copper and H_2_O_2_ in Ham's F12 at 4 °C for 4 days prior to the assay. The data are means (*n* = 3; ±SD) *p < 0.05; One-way ANOVA, Tukey's test). **(B)** Representative images of SH-SY5Y cells following incubation with 1 μM Aβ_1-42_-Hilyte-488, pre-treated with (i) NaOCl and (ii) copper as in (A) and 100 nM Lysotracker Red DND-99 at 37 °C for 1 h **(iii)** Chart shows quantified object area of each fluorescent signals and CoA normalised to total cell area in SH-SY5Y cells following treatment with NaOCl-treated Aβ_1-42_. Corresponding images are in (i). **(iv)** Chart shows quantified object area of each fluorescent signals and CoA normalised to total cell area in SH-SY5Y cells following treatment with copper-treated Aβ_1-42_. Corresponding images are in (ii). **(v)** Chart shows the relative CoA of the green and red fluorescent signals shown in (iii) and (iv). The colocalization of Aβ_1-42_ (green) in the lysosomes (red) were calculated as the colocalization area (CoA) of the green and red signals. The data are means (n = 27 areas from three wells; ±SD) ****p < 0.05; unpaired student's t-test). *Asterisks* denotes significant difference between NaOCl and Cu^2+^/H_2_O_2_. (For interpretation of the references to colour in this figure legend, the reader is referred to the Web version of this article.)

We next compared the internalisation of NaOCl-modified Aβ_1-42_ to that of Aβ_1-42_ exposed to metal-catalysed oxidative stress which has been shown to increase the neurotoxicity of Aβ_1-42_ via a mechanism involving dityrosine formation [[24], [25], [26], [27], [28]]. When the effect of pre-incubation with equimolar concentrations of copper-modified or NaOCl-modified Aβ_1-42_ on the toxicity of Aβ_1-42_ were directly compared, the results showed that copper-modified Aβ reduces the viability of SH-SY5Y cells by approximately 20%; however, under the same conditions NaOCl-modified Aβ_1-42_ did not induce neurotoxicity*,* as assessed by MTS assay (Fig. 7A). Analysis of the cellular uptake of the Aβ_1-42_ preparations using real-time imaging showed that SH-SY5Y cells preferentially internalise NaOCl-treated Aβ_1-42_ to lysosomes compared to copper-treated Aβ_1-42_, which is a potential route for the clearance of the peptide.

## 3 Discussion

This study contributes to the growing evidence that the functions of hypochlorite extend beyond its canonical antimicrobial activity. Specifically, we demonstrate that oxidation of Aβ_1-42_ by hypochlorite enhances its solubility by promoting the formation of higher order assemblies with decreased surface hydrophobicity compared to the unmodified peptide. Consistent with a model in which size and surface exposed hydrophobicity underpin the toxicity of misfolded protein assemblies [29], treatment with hypochlorite potently reduced the neurotoxicity of Aβ_1-42_ *in vitro*.

Our data demonstrate that treatment with hypochlorite has the general effect of reducing the total binding of Aβ_1-42_ to the surface of SH-SY5Y cells, however, this occurs in a multimodal manner whereby Aβ_1-42_ -associated cell surface fluorescence was lowest when treated with an intermediate amount of hypochlorite. This is most likely explained by the propensity of Aβ_1-42_ to form increasingly large assemblies when treated with higher concentrations of hypochlorite. It is a limitation of the current study that experiments involving hypochlorite-modified Aβ_1-42_ involved heterogeneous mixtures of Aβ_1-42_ assemblies and it is not possible to make comparisons between different Aβ_1-42_ preparations on a molar basis. Additional studies are required to identify specific receptors for hypochlorite-modified Aβ_1-42_ assemblies and characterise the physical properties of hypochlorite-modified Aβ_1-42_ that underpin interactions with receptor and/or interactions at the plasma membrane. Although internalisation to lysosomes is implicated in the neurotoxicity of soluble Aβ_1-42_ oligomers and dityrosine cross-linked Aβ [6,[30], [31], [32]], our results suggest that hypochlorite-modified Aβ_1-42_ is efficiently endocytosed to lysosomes without eliciting a corresponding neurotoxic effect. It is plausible that the self-limiting aggregation properties of hypochlorite-modified Aβ_1-42_ and the reduced surface exposed hydrophobicity of hypochlorite-modified Aβ_1-42_ assemblies are major factors contributing to this activity [29,30,33], however, further studies are required to precisely define the molecular mechanisms involved and the fate of internalised hypochlorite-modified Aβ_1-42_.

The exact concentration of hypochlorite generated in cerebral spinal fluid is not known, but myeloperoxidase has been measured in human cerebrospinal fluid (CSF) from cognitively normal donors at 101 ± 43 pg/ml with corresponding activity of 0.0292 ± 0.001 mU [34]. Although myeloperoxidase activity is much less in CSF than in human serum, human serum also contains high levels of albumin which acts as an antioxidant [35]. Additionally, using a highly selective, sensitive fluorescent probes, hypochlorite activity is detected in the brains of wild-type mice [36,37]. Considering that 3-chlorotyrosine, a specific marker of hypochlorite activity is present at around 2 nmol/mol tyrosine in post-mortem brain tissue from cognitively normal individuals [19] and is detected in CSF from children without central nervous system infection [38], available evidence supports that a basal level of hypochlorite-induced protein modification normally occurs in the human central nervous system. The results of *in vitro* experiments show that cortical neurons from rodents are resistant to cell death in the presence of up to 200 μM hypochlorite [39], however, further studies are needed to determine more precisely the point at which hypochlorite production becomes pathological.

Oxidised Aβ proteoforms are present in AD brain tissue and are detected in the CSF of individuals with and without AD [[40], [41], [42], [43]]. Consistent with a protective role for Aβ oxidation, amyloid deposits from AD senile plaques preferentially contains reduced Aβ compared to Aβ from deposits isolated from control brain tissue [44]. Consistent with the results of a prior study [20], our data show that Aβ_1-42_ is relatively resistant to hypochlorite-induced modification except for oxidation at a single site. This likely corresponds to the conversion of Met35 in Aβ_1-42_ to methionine sulfoxide [20]. Other abundant biological oxidants, such as hydrogen peroxide also preferentially react with Met35 of Aβ [25], however, comparatively hypochlorite reacts more readily [45,46]. Thus, even at low concentrations, hypochlorite is potentially an important chemical modifier of Aβ. Of note, the effects of hypochlorite and hydrogen peroxide on Aβ are distinctly different to those resulting from the reaction of Aβ with hydroxyl radicals (generated by metal-catalysed oxidation) that induce a broad range of modifications including intermolecular dityrosine bond formation, which is implicated in promoting Aβ-induced neurotoxicity [6,9,47]. As such, when considering the role that biological oxidants play in the ageing brain it is important to acknowledge the specific effects of biological oxidants rather than to refer collectively to oxidative stress.

Exacerbated hypochlorite production contributes to tissue damage in chronic or severe acute inflammation, including in AD where level of myeloperoxidase and 3-chlorotyrosine are estimated to increase 2-fold and 3-fold, respectively [36,48,49]. While brain-resident immune cells including microglia and astrocyte, and neurons have been reported to express myeloperoxidase [19,[50], [51], [52], [53], [54]], infiltrating neutrophils are likely an important source of myeloperoxidase in the disease [48,49]. Interestingly, SH-SY5Y cells have been shown to generate hypochlorite in response to Aβ_1-42_-Cu^2+^ [36] and treatment of rodent microglia with aggregated Aβ_1–42_ has been shown to induce MPO mRNA expression *in vitro* [52]. These observations suggest that hypochlorite production by brain-resident cells could be a response that aims to limit the accumulation of toxic Aβ assemblies in the brain. Consistent with the idea that non-pathological levels of hypochlorite production are important to protein homeostasis, the chaperone activity of alpha-2-macroglobulin, a biological carrier of Aβ, is dramatically enhanced following reaction with hypochlorite [16]. Also, the results of *in vitro* studies show that apolipoprotein E4, the strongest genetic risk factor for AD, is preferentially oxidised by hypochlorite compared to E3 and E2 [55]. Furthermore, oxidative modification of ApoE4, but not E3, promotes fibrillar aggregation of Aβ [56], which indicates that the effect of hypochlorite on Aβ/ApoE interactions is dependent on ApoE genotype.

A handful of studies have reported a link between polymorphism in myeloperoxidase and AD risk [52,[57], [58], [59], [60], [61]], however, a similar number of studies have not found an association [[62], [63], [64], [65]], nor has a genetic link been identified between myeloperoxidase and AD in genome-wide association studies. In the absence of a direct genetic link between mutations in myeloperoxidase and AD risk, it is important to identify the level of hypochlorite production that may be beneficial versus deleterious in the ageing brain. Most individuals with myeloperoxidase-deficiency (1 in 2000–4000 individuals in some populations [57]) do not experience serious adverse health consequences. It has been proposed that this is due to compensatory changes in other systems involved in the clearance of pathogens, such as increased proteolytic and phagocytic activity and an enhancement in adaptive immunity (reviewed in [59]). Consistent with this proposal, myeloperoxidase deficiency, is associated with increased risk of chronic inflammatory disease [57,58]. Interestingly, a relevant study reported 4 out of 92 myeloperoxidase-deficient individuals had AD, compared to none in the control group [58]. Further work is needed to characterise the effect of hypochlorite on Aβ *in vivo* and to determine, whether or not, compensatory mechanisms present in myeloperoxidase-deficient individuals also influence the toxicity and clearance of Aβ.

Data generated from a large number of studies suggest that the accumulation of Aβ is an early process that drives AD pathology, however, strategies targeting the clearance of Aβ have not proven robustly effective in the treatment of AD (reviewed in [66]). Considering that the accumulation of Aβ commences decades before the clinical features of sporadic AD emerge in afflicted individuals [67], it is plausible that targeting the clearance of Aβ much earlier than the clinical onset of AD may be needed to prevent the disease. Considering that exacerbated hypochlorite production is deleterious to human tissues, enhancing myeloperoxidase activity is unlikely to be a viable therapeutic strategy. However, small molecule drugs that target Met35 of Aβ are currently in development and have been shown to reduce Aβ-associated toxicity in animal models [68,69]. The result presented in this study shed new light on the likely importance of hypochlorite, and redox homeostasis more generally, on the toxicity and clearance of Aβ. More complete characterisation of the normal pathways for Aβ clearance and a greater understanding of the biological agents that influence these pathways could add to the framework for designing novel therapeutic interventions for preventing or treating AD.

## 4 Materials and methods

### 4.1 Materials

Chemicals and antibodies used were purchased from Sigma Aldrich unless otherwise stated.

### 4.2 Preparation of synthetic Aβ_1-42_

Unlabelled Aβ_1-42_, biotinylated Aβ_1-42_ and Hilyte-488 Fluor labeled-Aβ_1-42_ were solubilized according to the manufacturer's instructions (Anaspec Inc.,Fremont, CA, USA), except that NH_4_OH was replaced with an equimolar solution of NaOH. Aβ_1-42_ stock solutions were then snap-frozen in liquid nitrogen and stored at −80 °C for later use.

### 4.3 Treatment of Aβ_1-42_ with sodium hypochlorite (NaOCl)

Typically, Aβ_1-42_ (55 μM) was incubated with NaOCl (0–320 μM) in phosphate buffered saline (PBS), pH 7.4 overnight at ambient room temperature prior to experiments. When incubations differed from these standard conditions for a specific experiment, the details are provided in the relevant figure legend and in the relevant section of the Methods section.

### 4.4 Electrophoresis

Aβ_1-42_ was treated with NaOCl and separated by denaturing gel electrophoresis on a 10–20% Tricine gel (Life Technologies), according to the manufacturer's instructions using an XCell SureLock Mini-Cell electrophoresis apparatus (Invitrogen, VIC, AUS). Gels were stained using Instant Blue stain (Abcam; Melbourne, VIC, AUS) and imaged with Geldoc Go imager (Bio-Rad, NSW, AUS).

### 4.5 Western blot analysis

Following separation by electrophoresis, Aβ_1-42_ was transferred to PVDF membrane using an iBlot (Bio-Rad) and the membrane was blocked using 5% w/v skim milk powder in PBS, pH 7.4. Aβ_1-42_ was probed for using monoclonal anti-Aβ_1-42_ (Clone WO2) followed by an anti-mouse IgG-HRP conjugate (Life Technologies). Blots were imaged after exposure to enhanced chemiluminescence reagent (Bio-Rad, NSW, AUS) using a ChemiDoc MP Imaging system (Bio-Rad, NSW, AUS).

### 4.6 Filter-trap assay

Following treatment with NaOCl, Aβ_1-42_ samples were subjected to filter trap assay in order to estimate the amount of peptide present in the insoluble fraction. Briefly, Aβ_1-42_ samples were loaded into the wells of a Bio-Dot SF Microfiltration Apparatus (Bio-Rad) on to a 0.22 μm cellulose acetate membrane. Following extraction of the soluble fraction using a vacuum, the wells of the apparatus were washed three times using 0.01% (v/v) Triton-X in PBS. The membrane was blocked using 5% w/v skim milk powder in PBS and Aβ_1-42_ was probed for using monoclonal anti-Aβ_1-42_ (Clone WO2) followed by an anti-mouse IgG-HRP conjugate (Life Technologies). Blots were imaged after exposure to enhanced chemiluminescence reagent (Bio-Rad, NSW, AUS) using a ChemiDoc MP Imaging system (Bio-Rad, NSW, AUS). Densitometry analysis was performed to estimate the amount of Aβ_1-42_ retained on the membrane for each sample in triplicate using ImageJ software (NIH) [70]).

### 4.7 4,4′-dianilino-1,1′-binaphthyl-5,5′-disulfonic acid (bisANS) assay

Aβ_1-42_ was treated with NaOCl and the solutions supplemented with excess L-Met. For bisANS assay 10 μM Aβ_1-42_ was incubated with 20 μM bisANS in PBS for 5 min at 25 °C in the wells of a 384-well microtiter plate. BisANS fluorescence (Excitation 360 nm, Emission 502 nm) was then measured using a Clariostar platereader (BMG Labtech). The data shown is the average BisANS fluorescence of n = 10 samples and is adjusted for background fluorescence.

### 4.8 Thioflavin T (ThT) assay

Aβ_1–42_ (4–5 μM) was incubated at 28 °C with shaking in PBS ± NaOCl containing ThT (20–25 μM). The ThT fluorescence of the samples was continuously monitored using a Clariostar platereader with excitation and emission wavelengths of 440 nm and 480 nm (slit widths of 10 nm), respectively. The data shown is the mean fluorescence of triplicate samples and is adjusted for the background fluorescence. In additional experiments, Aβ_1–42_ was pre-treated with NaOCl in PBS for 1 h at ambient room temperature before being supplemented with excess L-Met (20 mM). A control sample of Aβ_1–42_ was incubated in the absence of NaOCl as described previously and supplemented with L-Met. Aβ_1–42_ preparations were then subjected to a ThT assay incubated at 37 °C under quiescent conditions, and monitored as described above. Half-time (_t1/2_) values are the time to reach half of the maximum ThT fluorescence, calculated after normalising the aggregation data for 3 independent assays.

### 4.9 Transmission electron microscopy (TEM)

Samples from the ThT kinetics assay (endpoints) were prepared for TEM on 400-mesh carbon film coated copper grids (EM resolutions Ltd.) and stained with 2% (w/v) uranyl acetate. Grids were imaged with a FEI Tecnai G2 transmission electron microscope (Cambridge Advanced Imaging Centre, University of Cambridge) and subsequent images analyzed with SIS Megaview II Image Capture system (Olympus).

### 4.10 Mass spectrometry (MS)

Aβ_1–42_ (31 μM) was incubated with NaOCl (0–360 μM) in PBS on ice for 1 h and the reaction was quenched using L-Met (20 mM). Electrospray mass spectrometry was performed on a Xevo G2 mass spectrometer and data was analyzed using MassLynx software (Waters UK, Elstree, Hertfordshire, UK) at the Department of Chemistry, University of Cambridge.

### 4.11 Size exclusion chromatography (SEC)

Aβ_1–42_ (66.67 μM) was incubated in the presence or absence of 133.33 μM NaOCl in PBS at ambient temperature for 1 h and then separated on a Superdex 75 Increase 3.2/300 column (GE Healthcare) using an AKTA Purifier FPLC (GE Healthcare) at a flow rate of 0.05 mL/min. The absorbance at 280 nm was used to monitor the elution of the peptide.

### 4.12 Cell culture

SH-SY5Y, a human neuroblastoma cell line, were grown in DMEM:F12 media supplemented with 10% (vol/vol) FBS, and routinely passaged using trypsin:EDTA. All cell culture reagents and media were obtained from GE Healthcare. All cell culture reagents and media were obtained from GE Healthcare. Cultured cells were maintained in a 37 °C incubator with 5% (v/v) CO_2_.

### 4.13 MTS assays

Aβ_1-42_ preparations were prepared by incubating Aβ_1-42_ (100 μM) in Ham's/F-12 media supplemented with 0–160 μM NaOCl at 4 °C for 4 days. In some experiments L-Met (200 μM) was added to the solutions at the end of this incubation period to quench any unreacted NaOCl. For MTS assays, SH-SY5Y were grown to ∼70% confluence in the wells of a 96-well plate using normal culture conditions. The standard media was then replaced with Neurobasal media containing 10 μM Aβ_1-42,_ B27 supplement and GlutaMAX™ (Life Technologies), according to the manufacturer's instructions. Vehicle control cells were treated with media supplemented with the appropriate volume of Ham's/F12 containing 0–160 μM NaOCl, 0–200 μM L-Met, B27 supplement and GlutaMAX™. cells were cultured for an additional 48 h before an MTS assay (CellTiter 96® AQueaous One Solution, Promega) according to the manufacturer's instructions. The absorbance of each well was measured at 490 nm using a SpectraMax D multi-mode microplate reader (Agilent, VIC, AUS). In additional experiments, we compared the toxicity of Aβ_1-42_ modified by metal-catalysed oxidation and NaOCl-treated Aβ_1-42._ In these experiments, Aβ_1-42_ (100 μM) was incubated in the presence of 160 μM Cu^2+^SO_4_ and 160 μM H_2_O_2_ or 160 μM NaOCl in Ham's F12 at 4 °C for 4 days. MTS assay was then performed, as described above, however, the final concentration of Aβ_1-42_ used in these experiments was 20 μM.

### 4.14 Incucyte assays

Cell viability was also measured in real-time using Cytotox Green reagent (Essen Bioscience) in an Incucyte SX5 (Sartorius, South VIC, AUS), according to the manufacturer's instructions. The preparation of Aβ_1-42_ and cell culture conditions were identical to those used for MTS assays, however, in these experiments we monitored the viability of SH-SY5Y cells for up to 95 h.

Incucyte analysis was also used to estimate the internalisation of Hilyte-488 fluor-labeled Aβ_1-42_ to lysosomes. To perform this Aβ_1-42_-Hilyte-488 (Anaspec) (50 μM) was pre-treated with 160 μM Cu^2+^SO_4_ and 160 μM H_2_O_2_ or 160 μM NaOCl in Ham's F12 at 4 °C for 4 days. SH-SY5Y cells were then cultured in the presence of 1 μM Hilyte 488 fluor-labeled Aβ_1-42_ in Neurobasal media containing B27 supplement and GlutaMAX™ (Life Technologies), according to the manufacturer's instructions, containing 100 nM Lysotracker Red DND-99. Images of the cells were taken after 1 h using an Incucyte SX5 (Sartorius).

### 4.15 Flow cytometry analysis

Biotinylated Aβ_1-42_ was treated with NaOCl in PBS using the standard method. SH-SY5Y cells were grown for 48 h without passage and detached from the flask using 5 mM EDTA in PBS. The cells were then washed twice with PBS and resuspended in Hanks Binding Buffer (HBB) (1 mM CaCl_2_, 0.5 M MgCl_2_–6H_2_O, 0.4 mM MgSO_4_.7H_2_O, 5 mM KCl, 0.4 mM KH_2_PO4, 4 mM NaHCO_3_, 138 mM NaCl, 0.3 mM Na_2_HPO4, 20 mM HEPES, 0.1% BSA), pH 7.4. Cells were then incubated with 0–5 μM Aβ_1-42_ in HBB (30 min on ice) followed by incubation with streptavidin-Alexa Fluor 488 (Life Technologies; 30 min on ice) in HBB, according to the manufacturer's instructions. The cell-associated Alexa Fluor 488 fluorescence was measured using a CytoFLEX S flow cytometer (Beckman Coulter) and the data were analyzed using Cytexpert software (Beckman Coulter). Propidium iodide was used to exclude non-viable cells from our analysis. The data shown is adjusted for the background fluorescence of cells treated with streptavidin-Alexa Fluor 488 alone.

### 4.16 Confocal microscopy

Aβ_1-42_-Hilyte-488 (Anaspec) (50 μM) was incubated ± 40 μM NaOCl in Ham's/F12 overnight at ambient room temperature. SH-SY5Y cells were seeded on poly-d-Lysine (100 μg/mL) coated-glass coverslips at 100,000 per well in DMEM/F12 supplemented with 10% FBS (Bovogen) and GlutaMAX™ (Life Technologies) and cultured overnight. Cells were then rinsed in serum-free DMEM prior to the addition of NB27 media containing 2.5 μM Aβ_1-42_-Hilyte-488 as described above. After 24 h in a humidified incubator at 37 °C and 5% (v/v) CO_2_, cells were rinsed in NB27 and incubated with 50 nM Lysotracker Red DND-99 (Life Technologies) and 5 μg/mL Hoechst 33342 (nuclear staining) for 30 min at 37 °C. Cells were washed twice in PBS and fixed in 4% paraformaldehyde in PBS (pH 7.4) for 10 min at ambient room temperature and then rinsed in PBS again. Cells were washed with water immediately prior to mounting using buffered glycerol (pH 8.6). Images were taken with a Zeiss LSM 880 Fast Airyscan Confocal Microscope using a 488 nm argon laser, 516 nm and 405 nm lasers. For the colocalization study, optical sections were 0.5 μm. Mander's coefficient values were calculated using Zeiss (Zen Black) software based on 5 images.

### 4.17 Endocytosis assay

Aβ_1-42_-Hilyte-488 (50 μM) was incubated with 160 μM NaOCl or Cu_2_SO_4_/H_2_O_2_ in Ham's/F12 or Ham's/F12 alone for 4 days at 4 °C. SH-SY5Y cells seeded at 15,000 cells/well in a 96-well plate were then treated with 1 μM of Aβ_1-42_-Hilyte-488 in NB27 containing 100 nM Lysotracker Red DND-99. Images of cells (9 images per well) were taken after 1 h using an Incucyte SX5 (Sartorius). Colocalization of Aβ_1-42_-Hilyte-488 with Lysotracker Red was calculated using Incucyte live cell analysis software.

## Declaration of competing interest

The authors declare that they have no known competing financial interests or personal relationships that could have appeared to influence the work reported in this paper.

## Data Availability

Data will be made available on request.
