# Supplementary material for: Hypochlorite-induced oxidation promotes aggregation and reduces toxicity of amyloid beta 1-42
Source: Redox Biol. 2023 May 13;63:102736. doi: 10.1016/j.redox.2023.102736 (PMC10209884; doi:10.1016/j.redox.2023.102736)
Supplement: Multimedia component 1 [file mmc1.docx]

**Supplementary Material**


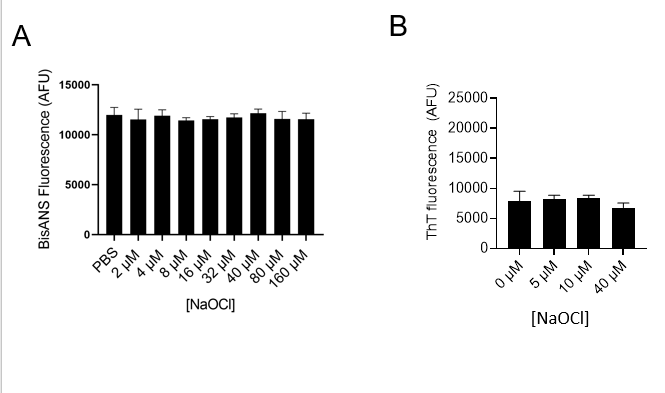


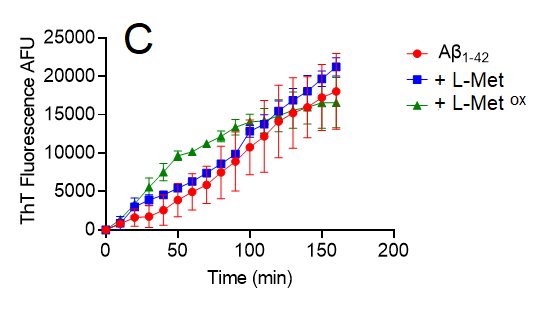


**Sup. Fig. 1 Quality control analysis showing the effect of NaOCl on BisANS fluorescence and ThT fluorescence (A)** Graph shows the Bis-ANS fluorescence *(Excitation = 360 nm, Emission = 502 nm)* 20 μM in PBS in the présence of NaOCl at the concentrations shown. The data are the mean (n=5; ±SD). No statistically significant differences were found between the BisANS fluorescence of the samples (One-way ANOVA, Tukey’s test). **(B)** Aβ_1-42_ (100 μM) was induced to form amyloid by incubation in PBS at 28 °C with orbital shaking in a Clariostar platereader. Aβ_1-42_ was then diluted to 5 μM in 0-40μM NaOCl in PBS containing 25 μM ThT and the ThT fluorescence was measured. Data is mean (n=4; ±SD). No statistically significant differences were found between the ThT fluorescence of the samples (One-way ANOVA, Tukey’s test). **(C)** Graph shows the ThT fluorescence of 5 μM Aβ_1-42_ in PBS and corresponding samples supplemented with 2 mM L-Met or 2 mM L-Met that had been pre-treated with NaOCl at a 1: 63 molar ratio (L-Met^ox^). All samples contained 25 μM ThT and were incubated at 37 °C with orbital shaking in Clariostar platereader. Data is mean (n=3; ±SD) of the background adjusted ThT fluorescence.


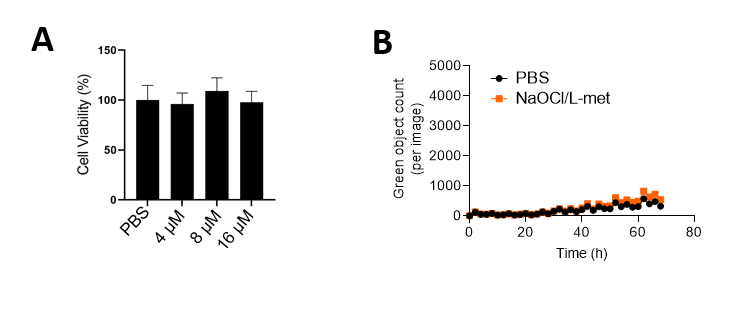


**Sup. Fig. 2 Quality control analysis showing the effect of incipient NaOCl and L-methionine on ThT fluorescence (A)** Chart shows the percent cell viability of SH-SY5Y cells as measured by MTS assay following treatment with NaOCl and L-Met at concentrations matched to the samples in Fig. 5A. The data are means (*n*=3; ±SD); One-way ANOVA, Tukey’s test. No significant differences among samples were found. **(B)** The graph shows the green object count per image in SH-SY5Y cells following treatment with NaOCl and L-Met alone at concentrations matched to the samples in Fig. 5B. The data are means (*n*=3; ±SD); unpaired student’s *t*-test. No significant differences among samples were found.


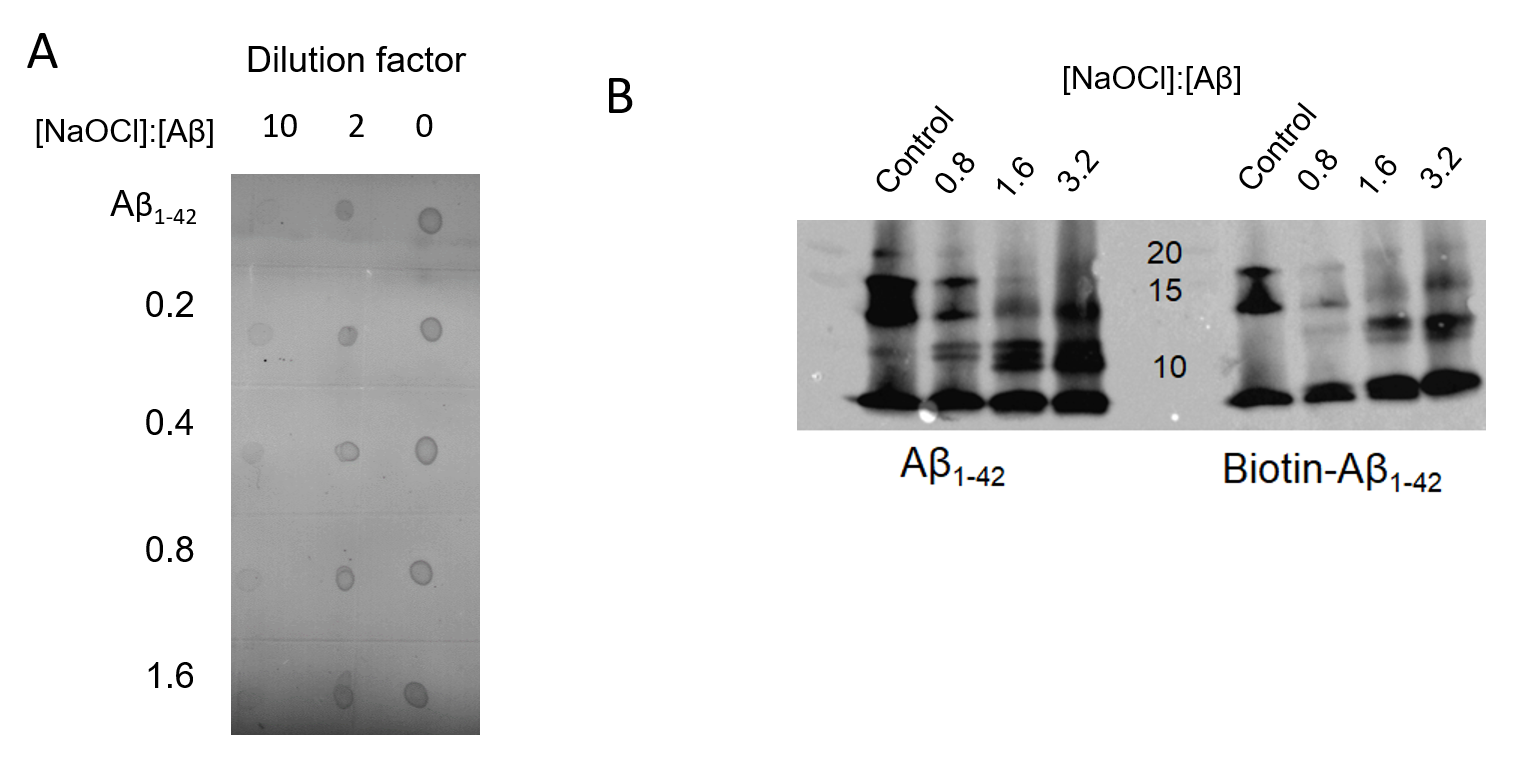


**Sup. Fig. 3 Quality control analysis showing the effect of NaOCl on biotinylated Aβ_1-42_** **(A)** Image of a dot blot showing the binding of streptavidin-Alexa-Fluor 488 to biotinyated Aβ_1-42_ following treatment with NaOCl at the molar ratios shown for 4 days at 4 °C. Streptavidin-Alexa-Fluor 488 was visualised using a Typhoon 9400 image scanner. **(B)** Image of a Western blot showing the migration of Aβ_1-42_ and biotinylated Aβ_1-42_ on a 4-12% bis-tris gel after treatment with NaOCl overnight at ambient room temperature at the molar ratios indicated.


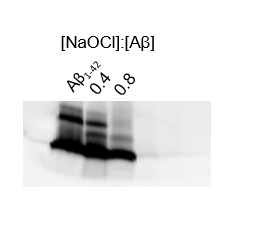


**Sup. Fig. 4 Quality control analysis showing the effect of NaOCl on Hilyte 488-Aβ_1-42_** Image of gel scan showing Aβ_1-42_ HiLyte-488 following treatment with NaOCl overnight at ambient room temperature. Aβ_1-42_ was separated on a 10-20% tris-tricine gel imaged directly using a Typhoon 9400 scanner (pixel = 500 um and power = 500 volt). Control samples are Aβ_1-42_ incubated in the absence of NaOCl. The molar ratio of NaOCl to Aβ in the remaining samples is indicated on the blot.
